# The effectiveness of rigid versus non-rigid dressings in reducing surgical site infections following major lower limb amputations: a systematic review

**DOI:** 10.1186/s13643-026-03180-3

**Published:** 2026-04-01

**Authors:** Jana Heinz, Zachary Moulder, Tim Staniland, Ross Lathan, George Smith, Ian Chetter

**Affiliations:** 1 Sheffield Teaching Hospitals NHS Foundation Trust, Sheffield, UK; 2 Hull University Teaching Hospitals NHS Trust Library Service, Hull, UK; 3 Academic Vascular Surgical Unit, Hull University Teaching Hospitals NHS Trust, Hull, UK; 4 Centre for Clinical Sciences, Hull York Medical School, Hull, UK

**Keywords:** Surgical site infection (SSI), Rigid dressings, Non-rigid dressings, Lower limb amputation, Postoperative complications

## Abstract

**Background:**

Surgical site infections (SSIs) are common complications following major lower limb amputation (MLLA), particularly among patients with diabetes and peripheral vascular disease. Postoperative dressing choice may influence SSI risk, yet the effect of rigid dressings (RDs) compared to soft dressings (SDs) on SSI risk is unknown. This review aimed to synthesise current literature analysing the effect of RDs versus SDs on SSIs and wound complications following MLLA.

**Methods:**

This study was conducted according to PRISMA and Cochrane guidelines. Medline, Embase, CINAHL, CENTRAL, and ClinicalTrials.gov were searched from inception to January 2025. Eligible studies included randomised controlled trials (RCTs), observational studies, and case series reporting SSI rates after MLLA managed with RD or SD. Due to the limited number of RCTs and substantial heterogeneity, a planned meta-analysis was not feasible, and a narrative synthesis was conducted. Risk of bias was assessed using RoB 2, ROBINS-I, and JBI tools, and certainty of evidence was evaluated with GRADE.

**Results:**

Ten studies (1 RCT, 6 observational, 3 case series) with 956 participants undergoing 1018 MLLAs were included. Reported SSI rates ranged from 0% to 77.8% in RD groups and from 13.5% to 65.9% in SD groups. The RCT demonstrated no significant difference in SSI rates between participants in the RD group (21.4%, 12/56) and the SD group (17.9%, 10/56), *p* = 0.47. Three observational studies reported lower SSI rates with RDs, while one reported higher rate. Wound dehiscence and revision surgery rates were generally lower in RD groups. Overall risk of bias was high across all studies, and no study reported SSIs as a primary outcome. Certainty of evidence was judged very low for all outcomes.

**Discussion:**

While some studies suggest a potential benefit of rigid dressings in reducing postoperative complications, the current evidence does not support the superiority of rigid or non-rigid dressings for SSI prevention. Findings are limited by substantial heterogeneity, confounding, and inconsistent reporting. High-quality prospective studies with standardised outcome measures are urgently needed to inform clinical practice.

**Systematic review registration:**

PROSPERO CRD42024607144.

**Supplementary Information:**

The online version contains supplementary material available at 10.1186/s13643-026-03180-3.

## Introduction

### Background

Major lower limb amputations (MLLA) are commonly performed for indications such as peripheral arterial disease, diabetes, or non-functional limbs secondary to trauma, malignancy, or congenital anomalies [1]. In many cases, particularly in patients with vascular disease or diabetes, amputation is preceded by chronic non-healing wounds that fail to respond to revascularisation, debridement, and multidisciplinary limb salvage strategies. In the USA alone, an estimated 50,000 to 60,000 individuals undergo major limb amputations annually, with peripheral arterial disease and diabetes mellitus reported as the leading underlying causes [2]. The prevalence of both conditions is expected to increase [3, 4]. The International Diabetes Federation projects that by 2045, one in eight adults will be living with diabetes, representing a 46% increase from current figures [4]. Similarly, the global burden of peripheral arterial disease is increasing, particularly among ageing populations and individuals with cardiovascular risk factors [3].

In the UK, the National Vascular Registry (NVR) shows a rising trend in annual MLLAs performed in vascular patients over the last 3 years from 3180 to 3688 [5]. Despite improvements in perioperative care, MLLA is associated with substantial morbidity and mortality. Reported mortality rates following MLLA vary widely [6], but the 2023 NVR reports a 30-day in-hospital mortality rate of 5.6% following MLLA [5].

Surgical site infections (SSIs) are a frequent and serious postoperative complication following MLLA [7]. SSIs are defined according to established criteria, commonly those of the Centers for Disease Control and Prevention (CDC), which require infection to occur within 30 days of surgery, involve superficial, deep, or organ/space tissues, and demonstrate features such as purulent drainage or microbiological confirmation [7]. The risk of developing SSIs is particularly elevated in individuals with diabetes and peripheral arterial disease, the two most common underlying conditions leading to amputation due to impaired wound healing, immunocompromise, and poor tissue perfusion [8]. A recent meta-analysis found an overall SSI incidence of 7.2% following MLLA, with through and below-knee amputations associated with higher infection rates (12.9% and 7.5%, respectively) compared to above-knee amputations (3.9%) [8].

Beyond clinical complications, MLLAs profoundly affect patients’ quality of life (QoL), often leading to significant physical disability, psychological distress, and social isolation [9–11]. Surgical site infections exacerbate this burden, prolonging hospitalisation and delaying rehabilitation and prosthetic fitting [12]. In recognition of their clinical importance, SSIs and wound healing outcomes have been prioritised in the development of core outcome sets for amputation studies, as identified through consensus by patients, carers, and clinicians [13].

The goals of early postoperative management are multifaceted and include promoting a favourable healing environment, controlling oedema and pain, preventing joint contractures, reducing time to prosthetic fitting, and supporting early mobilisation to enable faster return to daily activities [14]. Postoperative dressings play a key role in achieving these objectives. Common dressing modalities include soft dressings (SD), semi-rigid dressings (SRD), and rigid dressings (RD), each with distinct advantages and limitations [15].

The choice of postoperative dressing applied to the residual limb plays a crucial role in shaping recovery trajectories following major lower limb amputation. Soft dressings, such as elastic or crepe bandages, are widely adopted due to their affordability, ease of application, and accessibility across various healthcare settings [15]. However, they offer minimal protection from external trauma and are associated with limb oedema, and potential injuries to the residual limb due to inadequate compression or inconsistent application [15]. Rigid dressings, including both removable and non-removable variants, are increasingly advocated for their potential benefits in the immediate postoperative phase [14–16]. These dressings create a firm barrier around the residual limb, which may help reduce oedema, limit mechanical disruption, and promote faster wound healing [14–16]. They also provide mechanical stability that can prevent accidental trauma [15]. Removable rigid dressings (RRDs) offer the added advantage of easier wound inspection, while non-removable types may ensure more consistent compression but limit visual access to the surgical site [15]. Semi-rigid dressings, such as those incorporating Unna paste, represent an intermediate option, offering moderate support and compression while allowing for greater ease in application and removal compared to rigid options [15]. Despite their theoretical benefits, rigid dressings, particularly the non-removable type, are not without limitations. They can pose challenges for ongoing wound care, especially in cases where frequent inspection or dressing changes are required. The inability to routinely visualise the surgical site may delay the detection of complications such as wound dehiscence or infection, particularly in high-risk patients [1, 17].

While rigid dressings have gained popularity for their theoretical benefits, evidence supporting their role in preventing SSIs remains inconclusive [1, 15, 18, 19]. Several systematic reviews have examined a broad range of outcomes, including pain, stump volume, and time to prosthesis, but none have focused primarily on the incidence of SSI [1, 15, 18, 19]. SSIs are typically included as secondary outcomes, with inconsistent reporting of the effect of rigid dressings on SSI risk [1, 15].

This systematic review seeks to critically synthesise the existing evidence regarding the effect of rigid (RD) versus non-rigid (non-RD) postoperative dressings on the incidence of SSI and wound dehiscence following major lower limb amputation (MLLA), with the aim of informing future clinical practice and identifying priorities for high-quality research in this underexplored area. It is important to recognise that surgical site infections arise from complex interactions between patient-related factors, perioperative practices, postoperative wound care, environmental conditions, and rehabilitation processes. Dressing type therefore represents only one component within a multifaceted care pathway and cannot be considered in isolation. This complexity contributes substantially to the heterogeneity and confounding observed in existing studies.

## Methods

This study was designed and registered as a systematic review in accordance with PRISMA and Cochrane Handbook guidelines [20, 21]. A copy of the PRISMA statement is included in the supplementary information. The protocol for this review was prospectively registered with PROSPERO (ID: CRD42024607144) and has been submitted for publication; it is currently under peer-review.

### Objectives

#### Primary

- To evaluate the incidence of SSIs associated with rigid dressings versus non-rigid dressings in patients undergoing transfemoral, through-knee, and transtibial amputations.

#### Secondary

- To evaluate the incidence of wound dehiscence with rigid dressings versus non-rigid dressings in patients undergoing transfemoral, through-knee, and transtibial amputations.
- To assess postoperative recovery outcomes associated with rigid and non-rigid dressings, including overall wound problems, need for return to theatre, and mortality rates.

### Eligibility criteria

#### Types of studies

This study included RCTs, quasi-RCTs, observational studies, case reports and case studies that assess SSI rates in patients treated with rigid or non-rigid dressings. Observational studies were included to ensure a comprehensive view of literature given the anticipation of few RCTs. Studies were limited to English-language publications due to practical constraints, including the lack of access to translation services. There were no restrictions on sample size or study quality for studies meeting these criteria, allowing for a comprehensive literature assessment.

#### Inclusion criteria

- Participants: adults (≥ 18 years) who have undergone major lower limb amputation (transfemoral, through-knee, or transtibial).
- Intervention: rigid dressings (removable or non-removable) applied post-amputation. Immediate postoperative prosthesis (IPOP) was also included under the definition of rigid dressings, given its comparable application method and immobilisation function.
- Comparator: for interventional and observational studies with a comparison group, the comparator was participants who received any non-rigid postoperative dressing, including soft dressings (SD) and semi-rigid dressings (SRD).
- Outcomes: studies must report SSI rates.

### Information sources

Medline, Embase, CENTRAL, ClinicalTrials.gov and CINAHL databases were comprehensively searched from inception to January 2025**.**

In addition to database searches, the reference lists of included studies and excluded review articles were hand-searched for additional relevant studies, including those cited within them and their bibliographies. Newly identified articles were then uploaded to Covidence for further assessment.

### Search strategy

The search strategy was developed collaboratively with an information specialist (TS). A detailed search strategy, including an example from Embase, is presented in Additional file 1: Table 1.

### Selection process

Search results were uploaded to the Covidence systematic review software, which automatically removed duplicates [22]. Two researchers (JH and ZM) independently screened titles and abstracts for eligibility. Disagreements were resolved through consultation with a third senior researcher (RL). Articles deemed relevant underwent full-text assessment, again conducted independently by JH and ZM, with RL adjudicating any discrepancies. The study selection process was documented using the PRISMA flow diagram, detailing the number of search hits, duplicates removed, full texts reviewed and excluded (with reasons), and the final number of included studies [23].

### Data collection process

Data was collected independently by two researchers (JH and ZM) on two separate Microsoft Excel spreadsheets using a bespoke data extraction form. After data collection, the spreadsheets were compared, and any discrepancies were resolved by a third researcher (RL).

### Data items

Data were extracted on the following:

1. Study characteristics: year of publication, country, study design, sample size, length of follow-up, setting, and inclusion and exclusion criteria.
2. Participant characteristics: age, gender, smoking status, patient comorbidities including hypertension, diabetes, vascular disease, chronic foot wounds/ulcers, pre-operative infection, renal disease and immunosuppression, indication for operation, operation performed and perioperative use of antibiotics.
3. Descriptions of the dressing: RD, RRD, SD, SRD.
4. Primary outcome data: SSI incidence, method of SSI diagnosis (e.g. CDC criteria, ASEPSIS, or the Southampton scoring system, or other methods).
5. Secondary outcome data: wound dehiscence, defined as mechanical separation of wound edges, analysed separately from SSIs where reported; other wound complications described need and reason for return to theatre and mortality.
6. Details of the methodology relevant to risk of bias assessment such as study design, transparency, allocation, randomisation, blinding, heterogeneity between groups and analysis were also extracted at this stage.

To identify potential overlap, all included studies were screened for duplicate or overlapping data based on author names, location, sample size, and study period. All available results within each predefined outcome domain were systematically extracted using a standardised data collection form.

### Study risk of bias assessment

Two researchers (JH and ZM) independently assessed the methodological quality of included studies and the quality of evidence for key findings. Disagreements were resolved through consensus with a third researcher (RL). No automation tools were used in this process.

Risk of bias was evaluated using the Cochrane Risk of Bias 2 (RoB2) tool (for RCTs) [24], Risk of Bias in Non-randomised Studies of Interventions (ROBINS-I) tool (for observational studies) [25] and the Joanna Briggs Institute (JBI) Critical Appraisal Checklist for Case Reports (for case reports and case studies) [26].

Using these tools, the studies included were categorised into one of three groups relating to risk: “low risk,” “unclear risk,” or “high risk.” To visually summarise the risk of bias assessments, traffic light plots were generated for each study type using the corresponding assessment tool. Other bias categories were also assessed, with particular emphasis on baseline imbalance between participants, such as participant characteristics, including mean age, sex, or disease severity, as these may be related to outcome factors and can lead to bias in estimating the intervention effect.

Publication bias and selective outcome reporting were intended to be assessed using funnel plots for asymmetry. A minimum of 10 studies were required for this analysis, as advised by the Cochrane Handbook, to ensure sufficient power and reliability of the findings [20]. Fewer than 10 observational studies have been included in this review; therefore, the risk of bias due to missing results was evaluated qualitatively as part of the broader risk of bias assessment, particularly for outcomes not suitable for meta-analysis.

We anticipated that included studies would report outcome data relevant to the review objectives; however, in the event of missing or unclear data, study authors were to be contacted for clarification. No automation tools were used. In the presence of heterogeneity or incomplete reporting, potential selective outcome reporting and publication bias were explored descriptively.

### Data synthesis

#### Quantitative synthesis

Separate quantitative syntheses were planned for randomised controlled trials (RCTs) and non-randomised studies (NRS) to ensure methodological rigour and minimise bias associated with combining different study designs. However, due to substantial heterogeneity in study design, outcome definitions, and reporting across all included studies, as well as an insufficient number of comparable RCTs, quantitative synthesis was not feasible and a narrative synthesis was undertaken.

#### Narrative synthesis

A narrative synthesis was undertaken across all included studies. Studies were grouped by outcome domain and intervention type (rigid versus non-rigid dressings), and descriptive summaries will be used to highlight key findings and trends.

Variability in outcome definitions, measurement tools, and reporting methods was explicitly noted and discussed. Where necessary, absolute numbers were converted to percentages and vice versa to facilitate comparison between studies and ensure consistency in reporting across the synthesis.

### Certainty assessment

The certainty of evidence was assessed using the Grading of Recommendations, Assessment, Development, and Evaluation (GRADE) approach [27]. Certainty ratings were planned for each outcome to be presented in summary of findings (SoF) tables should studies permit. Where this was not feasible, the overall confidence in the findings was discussed narratively. Certainty assessments were based on the following criteria:

- Risk of bias: evaluated using the RoB2 tool for RCTs, ROBINS-I for non-randomised studies, and the JBI Critical Appraisal Checklist for case reports [24–26].
- Consistency: considered based on agreement of findings across studies and whether effect directions were aligned.
- Directness: assessed based on the applicability of study populations, interventions, and comparators to the review question.
- Precision: evaluated qualitatively by examining confidence intervals (when reported) and sample sizes across studies.
- Publication bias: assessed narratively by considering selective reporting and the likelihood of missing studies.

Two reviewers (JH and ZM) independently assessed certainty in the evidence, with disagreements resolved through discussion or consultation with a third reviewer (RL).

## Results

### Study selection

In total, 460 articles were identified from Embase (*n* = 303), Medline (*n* = 66), CINAHL (*n* = 48), CENTRAL (*n* = 22), and hand-searching (*n* = 21). After Covidence automatically removed 50 duplicates, 410 records remained for title and abstract screening. Following this stage, 330 articles were excluded, leaving 80 full-text articles for review. During the full-text analysis, five papers could not be retrieved despite multiple attempts to access them [28–32]. Due to their age (published between 1969 and 1978), these articles are not available in digital format [28–32]. The British Library, which holds an extensive archive, was contacted; however, its services remain unavailable following a cyber-attack in 2023, preventing further retrieval efforts. After full-text assessment, 65 of the 75 studies were excluded, resulting in 10 studies being included in the final review. The study selection process is also illustrated in the PRISMA flow diagram (see Additional file 1: Fig. 1).

Due to the insufficient number of RCTs and substantial heterogeneity among the included studies, a quantitative synthesis was not feasible, and all findings have been incorporated into a narrative synthesis. Studies were grouped by study design (RCTs, cohort studies, case series) and intervention type (rigid vs. non-rigid dressings). Differences in methodology, reported outcomes, and population characteristics were considered in the interpretation of findings. Where multiple study designs reported the same outcome, studies were narratively reviewed by type. If only a small number of studies reported an outcome, they were synthesised together as appropriate.

### Study characteristics

Ten studies published between 1968 and 2024 were included [33–42], spanning a period of major technological and clinical change in postoperative dressing materials and care pathways; one multicentre RCT [40], six observational studies (four retrospective cohort studies [34, 36, 38, 42], one prospective cohort study with a retrospective control group [37], and a retrospective case-control study [38]), and three case series (two prospective [33, 35] and one retrospective [41]). These studies span a period of major technological and clinical change in postoperative dressing materials, perioperative care pathways, and rehabilitation practices. A summary of this is demonstrated in Additional file 1: Table 2.

Details regarding adjustments to study data for synthesis are provided in Supplementary Material.

## Participants

Overall, a total of 1018 amputations were analysed, with individual study sample sizes ranging from 19 to 190 participants [33–42]. Participant characteristics were broadly consistent with the typical population undergoing major lower limb amputation, comprising predominantly middle-aged to elderly men with a high prevalence of cardiovascular risk factors such as smoking, diabetes, hypertension, and peripheral arterial disease. Baseline study and participant characteristics are demonstrated in Additional file 1: Table 2.

Two studies reported age as a median, ranging from 60.2 to 64 [33, 41]. Seven studies reported mean ages between 53 and 75.5 years [33–39, 42]. One study did not report participant age [40]. All included studies reported on gender, with participant numbers ranging from 0 to 103 women and 17–121 men per study [33–42]. Three studies reported smoking status, with 71% [35], 69% [42], and 62% of participants in the rigid dressing group being smokers [34].

### Comorbidities

Comorbidity profiles varied substantially across included studies, and baseline imbalances were common, especially given that most studies were non-randomised. Moreover, different studies reported different comorbidities, limiting the ability to compare populations and assess their potential impact on outcomes.

Diabetes mellitus was reported in nine studies, with eight showing that over 50% of participants had diabetes [33–37, 39, 41, 42]. The other paper reported a diabetes rate of just below 50%, suggesting a high prevalence across both groups [38]. Among the studies with a comparison group, they all reported a higher prevalence of diabetes in the rigid dressing group, which may have biased results against rigid dressings, given the known association between diabetes and impaired wound healing [34, 36–39].

Peripheral vascular disease was reported in nine studies demonstrating a prevalence in the rigid dressing group ranging from 33.7 to 100%. Among the rigid dressing groups, reported PVD rates ranged from 33.7% to 100%, while in soft dressing groups, PVD ranged from 22 to 100% [33, 35–42]. Several studies reported 100% prevalence of PVD in the rigid dressing group, whereas others, such as Mooney, reported only 33.7% [33, 35, 39–41]. Three comparative studies reported differences in the prevalence of PVD between groups. Schon et al. reported PVD in 37% of the rigid group compared to 22% in the soft dressing group [37]. Bikk et al. and Sarkar et al. both found slightly higher PVD rates in the soft dressing groups, 68.6% vs. 75% and 63% vs. 78%, respectively [36, 38].

Other comorbidities reported were hypertension [34, 36, 38, 42], pre-operative infection [33, 36, 37, 42], renal disease [34, 36, 38, 39, 42] and immunosuppression [38]. However, these were reported inconsistently across studies, limiting comparability between groups.

### Amputation level and indication

Eight studies included only below-knee amputations [33, 34, 36–41]. Two studies included below- and above-knee amputations [35, 42].

Six studies specified that amputations were performed secondary to vascular disease [33–35, 39, 40, 42]. Four studies reported infection or gangrene as the primary indication [33, 34, 37, 42].

### Perioperative antibiotics use

Only two studies reported on perioperative use [40, 41].

## Intervention—rigid dressing

### Rigid dressing

Eight studies used rigid, non-removable dressing [33–35, 37–41]. One study used a removable rigid dressing [36], while another one used an immediate postoperative prosthesis (IPOP) [42].

### Dressing change frequency

Among studies reporting dressing change protocols, five studies changed dressing weekly until healed (at around 3 weeks) before prosthesis fitting [34, 39–42]. The study by Bikk et al. changed dressings on week 1, 3 and 4 [38]. Condon et al. changed dressings at 2–3 weeks and again at 4–5 weeks before prosthesis fitting [33]. Schon et al. changed dressings daily until healing at around 2–3 weeks, then weekly [37]. Golbranson et al. initially changed dressing daily for the first 12 patients, before changing to as-needed changes [35].

### Timing of rigid dressing application

Rigid dressings were applied immediately following surgery in most studies [33–36, 38, 40–42]. One study further subdivided the rigid dressing group, with 57 patients receiving immediate fitting and 40 patients experiencing a delayed fitting a few days after surgery [39]. Another study fitted 20 patients with rigid dressing immediately and 10 patients within 5 days post-surgery [37].

## Comparator—soft dressing

Of the seven studies using a comparator group with soft dressing, three studies specified the use of elastic bandages, with one of them using additional knee immobilisers [37–39]. Four studies did not further specify the type of soft dressing used [34, 36, 40, 42]. In six of the included studies, soft dressings were applied immediately postoperatively as part of standard care in the control groups [34, 37–40, 42]. In the study by Van Velzen et al., although a conventional soft wound dressing was applied immediately, elastic bandaging was only initiated after suture removal [39]. Sarkar et al. did not specify the timing of soft dressing application in the control group [36].

## Outcomes

### SSI rates

None of the included studies reported a timeline for when surgical site infections (SSIs) occurred post-operatively, nor did they specify the diagnostic methods or scoring systems used. Consequently, SSIs could not be classified into short-term, medium-term, or long-term outcomes. Additionally, many studies did not report the severity of infections, limiting direct comparisons [33–42]. Results are summarised in Additional file 1: Table 3. There was a wide range of SSI rates in both the RD (0.0–77.8%) and SD (13.5–65.9%) groups.

#### RCT

The only RCT included in this review reported no significant difference in SSI rate between the RD group (21.4%, 12/56) and the SD group (17.9%, 10/56), *p* = 0.47 [40].

#### Observational studies

Three observational studies reported lower SSI rates in the RD group compared to the SD group [34, 37, 38].

In the study by Bikk et al., SSI occurred in 7 out of 70 (10%) of patients in the RD group and in 7 out of 52 (13.5%) of those in the SD group [38]. Ali et al. reported SSI rates of 7 out of 37 (18.9%) in the RD group compared to 9 out of 35 (25.7%) in the SD group [34]. Schon et al. found no SSIs in the 30 participants in the RD group compared to 4 out of 23 (17.4%) of patients with SSI in the SD dressing group [37].

Only one study reported a higher SSI rate in the RD group compared to the SD group [36]. Sarkar et al. found SSI rates of 42 out of 54 (77.8%) in the RD group and 27 out of 41 (65.9%) in the SD group [36].

Van Velzen et al. did not report SSI rates directly but instead categorised wound complications as superficial or deep, limiting direct comparability with other studies [39]. Wound complications were categorised as superficial, including local infection, superficial bleeding, and abrasion, or deep, including wound dehiscence, deep infection, and deep pressure ulcers. The study further divided the rigid dressing group into immediate and delayed fitting. Among all patients with RD, 30 out of 97 (30.9%) experienced superficial wound problems, with rates of 20 out of 57 (35.1%) in the immediate fitting group and 10 out of 40 (25%) in the delayed fitting group, compared to 12 out of 52 (23.1%) in the SD group. Deep wound problems were observed in 24 out of 97 (24.7%) of patients with RD, occurring in 10 out of 57 (17.5%) of the immediate fitting group and 14 out of 40 (35%) of the delayed fitting group, compared to 18 out of 52 (34.6%) in the SD group [39].

One study, despite including both study groups in its design, only reported SSI rates for the RD group, which was 2 out of 69 (2.9%) [42]. Of these two cases, one required antibiotic treatment, while another progressed to sepsis, resulting in patient death [42].

#### Case series

Among the three-case series, reported SSI rates varied widely from 2.6% to 25% [33, 35, 41]. Condon and Jordan reported an SSI rate of 2.6% (1/38) with RD [33]. Mooney et al. found an SSI rate of 8.4% (16/190) with RD, of which eight cases were superficial infections that delayed healing but did not prevent prosthetic fitting, while another eight were deep infections that progressed to osteomyelitis, necessitating revision surgery [41]. The case series by Golbranson et al. reported an SSI rate of 25% (5/20) with RD, classifying one infection as major and four as minor, though no further classification details were provided [35]. The heterogeneity in case series methodology and reporting limits their comparability with the observational studies.

Overall, most comparative studies suggest that rigid dressings may be associated with lower SSI rates; however, inconsistencies in reporting methods and variability in individual study findings prevent definitive conclusions [33–42].

### Wound dehiscence

Wound dehiscence was analysed as a distinct postoperative complication, separate from surgical site infection, although some studies reported overlapping wound-related outcomes. Wound dehiscence was reported in four studies [34, 35, 37, 38]. One case series found a 10% (2/20) incidence of wound dehiscence with RD, with one case classified as minor and the other as major; however, no criteria were provided for this classification [35].

Among the three studies with a comparison group, two reported lower wound dehiscence rates in the RD group [37, 38]. In the study by Bikk et al., wound dehiscence occurred in 7.1% (5/70) of patients in the RD group compared to 23% (12/52) in the SD group [38]. Schon et al. reported no cases of wound dehiscence in the 30 participants in the RD dressing group, while 13% (3/23) of participants in the SD group developed dehiscence [37]. In contrast, Ali et al. found slightly higher rates of wound dehiscence in the RD group (29.7%) compared to the SD group (25%), but only reported percentages [34].

While RD appear to reduce wound dehiscence in most studies, Ali et al. reported higher rates, highlighting the need for further investigation into potential confounders [34, 35, 37, 38]. The slightly higher rate of wound dehiscence in the RD group may be influenced by a high burden of comorbidities such as diabetes and chronic wounds at baseline, which were prevalent in this cohort [34].

### Other wound complications

Six studies reported additional wound-related complications [33–35, 37, 38, 42]. A case series by Condon et al. identified a 21.1% (8/38) incidence of superficial necrosis (21.1%) with RD, with four resulting in eschar formation and uncomplicated healing, while the remaining four experienced slightly prolonged healing (5–6 weeks) [33]. Three cases (7.9%) had delayed wound healing, including two cases of skin necrosis that sloughed, leaving a granulating ulcer, and one major wound infection. Additionally, two patients developed draining sinuses (5.3%) at 2 and 5 months post-operatively, while four required further amputation due to stump necrosis [33]. Golbranson et al. also reported 4 out of 20 patients (20%) developed skin necrosis, two cases of minor skin necrosis and two cases of major skin necrosis, along with 13 cases (10.5%) of delayed wound healing [35].

Among the comparative studies, Bikk et al. found wound necrosis in 11.4% (8/70) of participants in the RD group and in 13.5% (7/52) of participants in the SD group [38]. Stump injury was observed in 5.7% (4/70) of participants in the RD group and 11.5% (6/52) of participants in the SD group, suggesting a possible protective effect of rigid dressings against mechanical trauma [38]. Ali et al. reported skin breakdown separate from the incision in 18.9% (7/37) of participants in the RD group and 27.9% (9/35) of participants in the SD group, further indicating a protective role of rigid dressings [34]. Schon et al. described blisters in 6.7% (2/30) and a pretibial ulcer in 3.3% (1/30) of participants in the RD group [37]. In contrast, non-healing suture lines and partially healed suture lines with eschar formation were seen in 21.7% (5/23) and 13% (3/23) SD group participants, respectively [37]. Lastly, Folsom et al. reported one case (1.5%) of traumatic stump disruption in the RD group, caused by a patient falling [42]. The wound was subsequently managed with secondary closure and conventional treatment [42].

### Re-operation

All included studies reported the need for re-operation [33–42].

#### RCT

Revision to an above-knee amputation was required in 2.6% (2/56) participants in the RD group and 5.4% (3/56) participants in the SD group. The reasons for re-operation were not reported [40].

#### Observational studies

Six observational studies reported higher revision surgery rates in the SD group compared to the RD group [34, 36–39, 42]. Bikk et al. found a higher-level amputation was necessary in 7.1% (5/70) RD participants and 9.6% (5/52) SD participants [38]. Sarkar et al. reported a more pronounced difference, with a re-amputation incidence of 9.3% (5/54) in the RD group versus 41.5% (17/41) in the SD group [36]. Ali et al. similarly observed lower re-operation rates in the RD group (5.4%) compared to the SD group (27.6%) [34]. Schon et al. found no revision surgeries in the RD group while 43.5% (10/23) of limbs in the SD group required revision surgery [37]. These 10 re-operations were needed for 8 patients (2 patients had bilateral amputations) [37].

Van Velzen et al. provided additional insight by subdividing the RD group into immediate and delayed fitting. A higher level amputation was less commonly required in the immediately fitted RD group (5.3%, 3/57) than in the delayed RD fitting group (25%, 10/40) or the SD group (17%, 9/52) [39]. One study, which analysed only the RD group, reported that higher-level amputation was required in 1.5% (1/69) participants [42].

#### Case series

Among the three-case series, Condon and Jordan reported that 7 out of 38 patients (18.4%) required re-operation, including one for removal of skin and fascial sutures, two for probing of sinuses and myodesis suture removal, and four requiring above-knee amputations [33]. Golbranson et al. found that 1 out of 20 (5.0%) required a more proximal amputation [35]. Mooney et al. reported that 8 out of 190 (4.2%) underwent higher-level amputation due to post-operative infections [41].

Overall, the majority of studies reported a lower need for revision surgery in the rigid dressing group compared to the soft dressing group [34, 36–38, 40]. The largest differences were observed in observational studies, where some reported markedly reduced re-operation rates with rigid dressings [34, 36–38]. However, variations were noted, particularly in Van Velzen et al., where delayed rigid dressing fitting was associated with a higher rate of above-knee amputation [39]. Rigid dressings were generally associated with a lower need for re-operation, particularly when applied immediately post-operatively [39].

### Mortality

Six studies reported mortality, though none specified the timeline postoperatively [33, 34, 37, 39, 40, 42].

The only RCT included in this review reported a mortality of 25.0% (14/56) in the RD group and 17.9% (10/56) in the SD group [40].

Among the observational studies, Ali et al. reported no deaths in the RD group, while 1 of 35 patients (2.9%) in the SD group died due to respiratory sepsis [34]. Folsom et al. observed a mortality rate of 5.8% (4/69) in the RD group, with three deaths due to cardiac events and one from postoperative sepsis following an SSI. Mortality rate in the SD group was not analysed [42]. Van Velzen et al. reported in-hospital mortality rates of 28.1% (16/57) in the immediate RD group, 32.5% (13/40) in the delayed RD group and 23.1% (12/52) in the SD group [39].

Among the case series, Condon et al. reported a mortality rate of 10.5% (4/38), with causes including two cerebrovascular accidents (CVA), one myocardial infarction (MI), and one unknown [33]. Golbranson et al. found a mortality rate of 35% (7/20) with deaths attributed to five coronary thromboses, two CVAs, and one case of chronic emphysema [35]. Mooney et al. reported one death out of 190 (0.5%) due to an MI [41].

The relationship between dressing type and mortality remains unclear due to inconsistencies in reporting and the absence of adjustments for baseline comorbidities. While some studies suggest a potential increase in mortality with delayed rigid dressing application, overall findings are highly variable [39]. A summary of findings (Additional file 1: Table 4) presents the overall findings and certainty of the evidence across all outcomes.

## Risk of bias in studies

Overall, the included studies demonstrated high to critical risk of bias, with observational studies contributing the greatest limitations [33–42].

The single randomised controlled trial was judged to be at high risk of bias, while all observational studies were assessed as having critical overall risk of bias, primarily due to confounding, selection bias, and limitations in outcome measurement [34, 36–40, 42]. Case series studies were similarly judged to be at high risk of bias [33, 35, 41].

Detailed domain-level assessments for each study are presented in Additional file 1: Figs. 2–4.

## Certainty of evidence

Certainty of evidence for each outcome was assessed using the GRADE approach, which considers five domains: risk of bias, inconsistency, indirectness, imprecision, and publication bias. Given the heterogeneity in study design, outcome definitions, and the predominance of observational studies, the findings could not be pooled quantitatively. A summary of findings (SoF) table was therefore developed to present the effect estimates and certainty of the evidence across outcomes.

### Surgical site infection (SSI)

The overall certainty of evidence for SSI is *very low*. This rating reflects a critical or serious risk of bias across all included studies, inconsistent definitions and time points for SSI, and imprecision stemming from small sample sizes and a wide range of reported infection rates (0–77.8%) [33–42]. Only one randomised controlled trial [40] was available, and it was judged to be at high risk of bias. Although observational studies generally favoured RD, the lack of adjustment for confounding factors substantially limits confidence in these findings [34, 36–39, 42].

### Wound dehiscence

The certainty of evidence is *very low*, primarily due to serious risk of bias, inconsistency, and indirectness. While some observational studies reported lower dehiscence rates with RD [37, 38], another reported higher rates [34]. Definitions and assessment methods for dehiscence were either inconsistent or poorly described [33–42].

### Other wound complications (e.g. necrosis, blistering, delayed healing)

The certainty of evidence is *very low,* driven by the descriptive nature of reporting, lack of standardised outcome classification, and variation in how complications were defined and measured. Findings were conflicting, with no consistent methodology across studies [33–35, 37, 38, 42].

### Re-operation rates

The certainty of evidence is *very low*. While most comparative studies suggested fewer re-operations in the RD group, none were adequately powered or adjusted for key confounders such as comorbidities or surgical technique [34, 36–39]. One RCT addressed this outcome but had a small sample size and a high risk of bias [40].

### Mortality

The certainty of evidence is *very low*. Although six studies reported mortality, none adjusted for confounders, and reporting timeframes were inconsistent or not specified [33–35, 39, 41, 42]. The only RCT reported higher mortality in the RD group but did not provide contextual explanations or data [40].

Across all outcomes, the main reasons for downgrading the certainty of evidence were:

High or critical risk of bias in all included studies.

- Serious inconsistency in the direction and magnitude of effect estimates
- Serious indirectness due to unstandardised and poorly reported outcome definitions
- Serious imprecision, including small sample sizes and wide confidence intervals (where reported)
- Suspected publication bias, although not formally assessed due to the limited number of studies

## Discussion

### Interpretation of evidence

The included studies suggest a possible trend favouring rigid dressings (RDs) in reducing surgical site infections (SSIs) and revision surgeries following major lower limb amputation. This trend is primarily driven by observational studies, which generally reported lower SSI and revision rates in RD groups compared to soft dressing (SD) groups. For instance, Bikk et al. and Ali et al. reported reduced SSI rates in the RD group compared to SD, while Schon et al. observed no SSIs in the RD at all [34, 37, 38]. In contrast to the general trend, Sarkar et al. found higher SSI rates in the RD group, though this study involved a high-risk cohort and lacked detailed adjustment for confounding variables [36]. Importantly, these findings do not establish a causal relationship between dressing type and infection outcomes.

The sole RCT included in this review did not demonstrate clear superiority of one dressing type and was underpowered [40]. The case series, while contributing descriptive insights, were not intended for direct comparative analysis.

In summary, while some findings suggest potential advantages of RDs, the overall evidence remains tentative. The wide variation in SSI incidence reported across studies, from 0% to 77.8%, highlights the urgent need for standardised definitions and robust comparative methodologies in future research [33–42].

### Limitations of the evidence

The available evidence is subject to several important limitations. All included studies were assessed as having a high or critical risk of bias for the outcome of surgical site infection [33–42]. Despite this, all studies were retained to ensure that the review captured the full scope of available evidence in this sparsely researched area.

Substantial heterogeneity was observed across studies in terms of study design, patient populations, outcome definitions, and follow-up durations. Across all studies, the timing of SSIs was not reported, and no study specified standardised diagnostic criteria (e.g. CDC or ASEPSIS), allowing for potential subjective classification and inter-observer variability [33–42]. Infections were not graded by severity; only one study reported both a mild infection treated with antibiotics and a severe case progressing to sepsis [42].

Importantly, none of the included studies investigated SSIs as a primary outcome [33–42]. In several cases, reporting was incomplete or unclear, and only subsets of participants from the original study populations were included in analyses [34, 35, 40].

Several studies lacked clarity in reporting: some did not provide absolute values, only percentages, complicating interpretation and verification; others only reported outcomes for one treatment group despite including both [34, 42]. Studies that included mixed amputation levels or required subgroup analysis due to incomplete reporting reduced the effective sample size and interpretability of outcomes [35, 42]. In addition, outcomes such as wound dehiscence, necrosis, and re-operations were inconsistently reported and often lacked standardised definitions, complicating cross-study comparisons. In particular, wound dehiscence represents a mechanical complication distinct from infection, and its relationship with SSI risk could not be reliably assessed due to inconsistent reporting. Mortality data were also commonly reported without specific post-operative timeframes or adjustments for potential confounders, such as comorbidities, which may have influenced observed group differences [33, 34, 37, 39, 40, 42].

Important confounding variables known to influence SSI risk, including perioperative antibiotic use, smoking status, and comorbidities, were inconsistently reported and rarely adjusted for. While most studies provided some information on comorbidities such as diabetes, hypertension, immunosuppression, renal disease, and peripheral arterial disease, these variables were not consistently accounted for in analyses [33–42]. For example, Ali et al. reported higher wound dehiscence rates in the RD group; however, this group also had a greater burden of chronic wounds and comorbidities at baseline, which were not controlled for [34].

Many additional contributors to infection risk were poorly reported or entirely absent, including operating theatre sterility practices, postoperative wound care education, availability of wound care resources, environmental hygiene, and adherence to infection prevention protocols. These unmeasured variables introduce substantial residual confounding and further limit the ability to attribute infection outcomes to dressing type alone.

In all studies, assessors and patients were aware of the intervention, introducing potential performance and detection bias [33–42]. Additionally, details regarding dressing application, provider training, and standardisation of technique were largely absent. Only one study reported structured training for dressing application [40]. Variability in application technique may influence outcomes such as pressure injury, wound breakdown, and infection risk [43].

Frequency of dressing changes also varied across studies [33–42], which may have influenced both detection and reporting of SSIs. Infrequent changes may delay recognition of infection, while frequent changes may lead to earlier detection and reduced apparent differences between groups.

Several included studies were dated, some from as early as 1968, 1969, 1976, and 1992, raising concerns about the quality of study conduct, reporting, and methodological rigour [33, 35, 41, 42]. The inclusion of studies spanning more than five decades introduces challenges in interpretation due to evolving clinical practice, dressing technologies, and perioperative care pathways. While necessary to capture the full scope of available evidence, this limits comparability across eras.

### Historical evolution

In addition to the limitations outlined above, it is important to consider the evolution of dressing technologies over time. Early studies included in this review primarily utilised traditional postoperative dressings and immobilisation techniques that limited access to the residual limb [39]. More recent practice incorporates removable rigid systems and structured rigid dressings with enhanced material properties that facilitate regular monitoring and individualised management [14]. These developments in dressing technology and clinical practice reflect broader changes in amputation care over recent decades and may influence wound healing trajectories, rehabilitation, and complication detection, and should be considered when interpreting outcome differences across studies conducted in different eras.

### Limitations of the review process

This review has several methodological limitations. During full-text screening, five eligible studies could not be retrieved due to their age and unavailability, despite attempts through sources including the British Library [28–33]. This may have introduced selection bias.

Due to the limited number of available studies, all study designs were included to provide a comprehensive overview of the evidence. Although necessary, this approach increases the overall susceptibility to bias, particularly through the inclusion of observational studies and case series.

Although a quantitative synthesis was planned, this was not feasible due to substantial heterogeneity in study design, outcome definitions, and reporting standards, as well as the limited number of comparable studies. As a result, a narrative synthesis was undertaken, which is inherently more susceptible to subjective interpretation. Only one RCT was identified, precluding meta-analysis [40].

The limited number of studies also prevented formal assessment of publication bias, such as funnel plot analysis. In addition, selective reporting bias cannot be excluded, particularly given the scarcity of high-quality interventional studies.

### Implications of the results for practice, policy, and future research

This systematic review highlights the limited and low-certainty evidence available on the association between postoperative dressing type and outcomes following major lower limb amputation. Across all outcomes, the certainty of evidence was rated as very low, reflecting the predominance of observational studies, heterogeneity in outcome definitions, and small sample sizes.

Existing studies are predominantly observational, with significant methodological limitations, inconsistent outcome definitions, and inadequate adjustment for confounding factors. As a result, definitive conclusions regarding the comparative effectiveness of dressing types in preventing SSIs cannot be drawn. By explicitly documenting methodological weaknesses and sources of bias, this review provides important context for interpreting current evidence, clarifies why definitive recommendations are not currently possible, and identifies key priorities for improving future research quality.

The most important implication of this review is the clear need for methodologically rigorous future research. Well-designed prospective studies, particularly randomised controlled trials, are required to evaluate dressing strategies using standardised definitions of SSIs, appropriate follow-up periods, and adequate control of confounding variables. Improved reporting standards and consistency in outcome measurement are essential to enable meaningful comparison across studies and to inform clinical guidelines and policy.

In the absence of definitive comparative evidence, postoperative dressing selection should be individualised based on patient comorbidities, wound characteristics, rehabilitation goals, and local clinical expertise.

## Supplementary Information

Supplementary Material 1: Table 1: Example Embase search strategy from 1974 to 2024 January 31. Fig. 1 PRISMA flow diagram. Table 2: Study and participant characteristics. Table 3: Clinical outcomes stratified by dressing type. Includes SSI rates, wound dehiscence, return to theatre, and mortality. Table 4: Summary of findings: rigid versus soft dressings for major lower limb amputation. Fig. 2 Risk of bias assessment of the included RCT risk using the RoB 2 tool. Fig. 3 Risk of bias assessment of included observational studies using ROBINS-I tool. Fig. 4 Risk of bias assessment of included case series using a modification of the Joanna Briggs Institute critical appraisal tool.

Supplementary Material 2. PRISMA.

## Data Availability

The datasets supporting the conclusions of this review are included in Additional file 1. The full data collection form used during the current study is available from the corresponding author (Jana Heinz) on reasonable request.
