# Supplementary material for: The effectiveness of rigid versus non-rigid dressings in reducing surgical site infections following major lower limb amputations: a systematic review
Source: Syst Rev. 2026 Apr 1;15:163. doi: 10.1186/s13643-026-03180-3 (PMC13170014; doi:10.1186/s13643-026-03180-3)
Supplement: Supplementary file 1 — Supplementary Material 1: Table 1: Example Embase search strategy from 1974 to 2024 January 31. Fig. 1 PRISMA flow diagram. Table 2: Study and participant characteristics. Table 3: Clinical outcomes stratified by dressing type. Includes SSI rates, wound dehiscence, return to theatre, and mortality. Table 4: Summary of findings: rigid versus soft dressings for major lower limb amputation. Fig. 2 Risk of bias assessment of the included RCT risk using the RoB 2 tool. Fig. 3 Risk of bias assessment of included observational studies using ROBINS-I tool. Fig. 4 Risk of bias assessment of included case series using a modification of the Joanna Briggs Institute critical appraisal tool. [file 13643_2026_3180_MOESM1_ESM.docx]

# Supplementary Material

**Manuscript Title:**

The effectiveness of rigid versus non-rigid dressings in reducing surgical site infections following major lower limb amputations: a narrative review

**Authors:**

Jana Heinz^1*^, Zachary Moulder^2^, Tim Staniland^3^, Ross Lathan^4^, George Smith^5^ Ian Chetter^6^

**Corresponding Author:**

### Jana Heinz – jana.heinz1@nhs.net**Supplementary methods**

**Adjustments to study data for synthesis**

Some included studies required modifications in the number of participants analysed or in the way outcome data were extracted, based on reporting limitations. Where binary outcomes were reported as absolute numbers, percentages were calculated to facilitate comparisons. Conversely, in Ali et al., only percentages were reported (35). While the paper stated that 37 patients were in the rigid dressing group (35 with full data) and 35 in the soft dressing group, no absolute numbers were provided for specific outcomes. Attempts to reverse-calculate absolute values led to non-integer results, suggesting discrepancies in reporting. Therefore, only the reported percentages were used (35).

Woodburn et al. initially reported enrolling 78 patients in the rigid dressing group and 76 in the soft dressing group (total n = 154) (41). Of these, 96 patients proceeded to casting, 16 were deemed unfit for prosthesis, and 112 completed the trial. However, outcome data in the results section were consistently presented as “out of 56” per group, suggesting that only 56 participants in each group were included in the final analysis. To ensure accuracy and consistency, this review uses n = 56 per group when reporting data from this paper (41).

Golbranson et al. reported a larger cohort; however, surgical site infection (SSI) rates were only provided for patients undergoing amputation due to vascular disease (36). As such, our analysis was restricted to this subgroup, initially narrowing the cohort to 24 amputations. The study also included various amputation types, including foot and Syme amputations, which did not meet the inclusion criteria for major lower limb amputations. These cases were independently reviewed and excluded by two reviewers (JH and ZM), resulting in a final total of 20 eligible major lower limb amputations included in this analysis (36).

### Supplementary Tables and Figures

**Table 1:**  *Example Embase search strategy from 1974 to 2024 January 31.*

| Line No. | Search Query | # Results |
| --- | --- | --- |
| 1 | exp below knee amputation/ | 4131 |
| 2 | Transtibial Amputation*.mp. | 1324 |
| 3 | below knee amputation*.mp. | 4806 |
| 4 | exp leg amputation/ | 17420 |
| 5 | 1 or 2 or 3 or 4 | 18527 |
| 6 | SSI.mp. | 17968 |
| 7 | Surgical Site Infection*.mp. | 30553 |
| 8 | exp surgical infection/ | 75047 |
| 9 | exp postoperative infection/ | 117062 |
| 10 | exp postoperative complication/ | 1073399 |
| 11 | exp postoperative care/ | 120327 |
| 12 | exp wound healing/ | 225447 |
| 13 | surgical wound infection.mp. | 2306 |
| 14 | exp infection rate/ | 44416 |
| 15 | Infection Rate*.mp. | 70959 |
| 16 | 6 or 7 or 8 or 9 or 10 or 11 or 12 or 13 or 14 or 15 | 1420179 |
| 17 | exp wound dressing/ | 38904 |
| 18 | exp "bandages and dressings"/ | 84396 |
| 19 | dressing*.mp. | 68831 |
| 20 | 17 or 18 or 19 | 109626 |
| 21 | 5 and 16 and 20 | 358 |

**Legend:** *Search strategy used to identify relevant studies in Embase, developed in collaboration with information specialist TS.*

**Figure 1:** *PRISMA flow diagram*


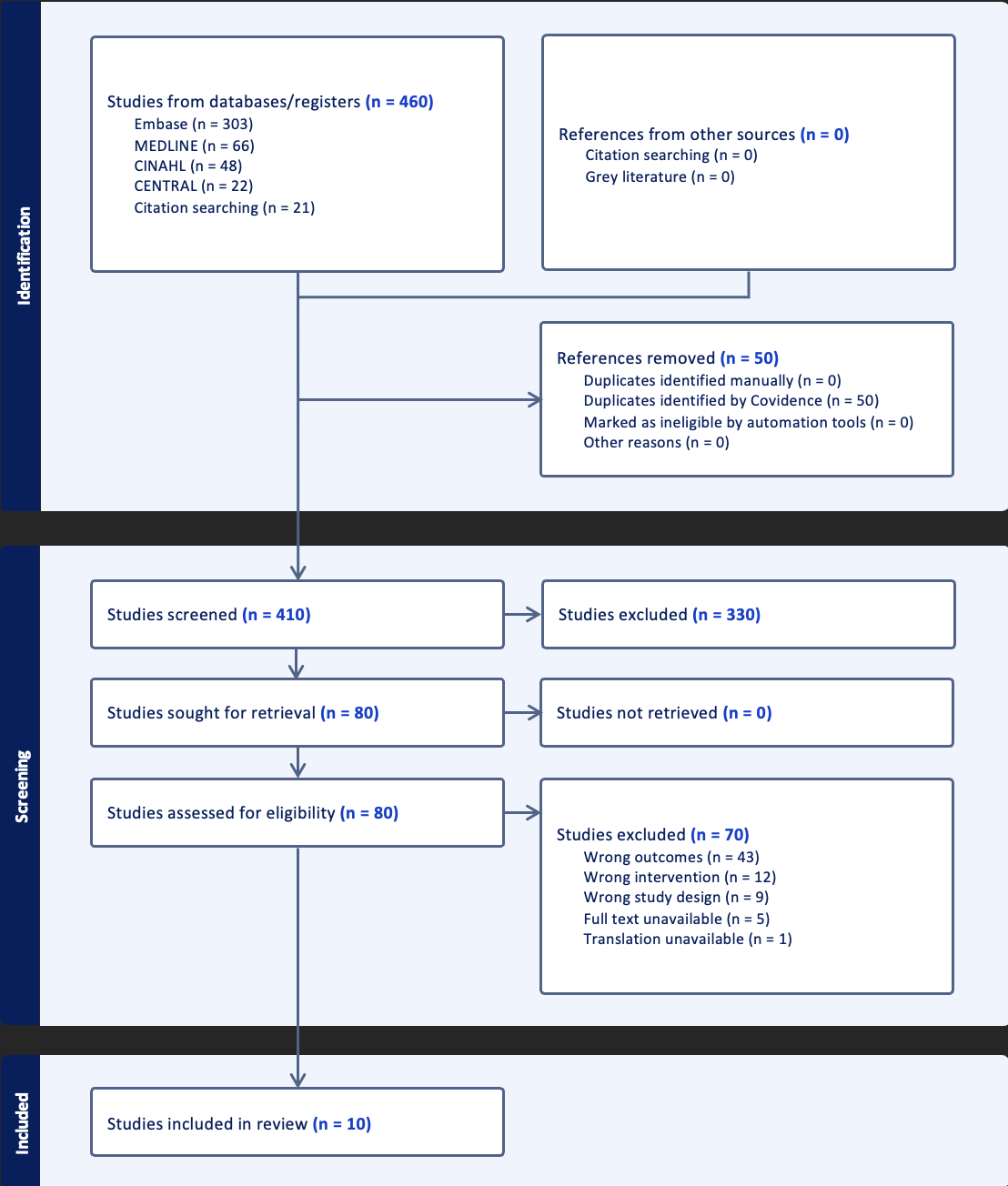


**Table 2:** *Study and participant characteristics*

| **First Author** | **Year** | **Country** | **Study Design** | **RD: Age** | **SD: Age** | **RD: Gender** | | **SD: Gender** | | **RD: DM** | **SD: DM** | **RD: PVD** | **SD: PVD** |
| --- | --- | --- | --- | --- | --- | --- | --- | --- | --- | --- | --- | --- | --- |
|  |  |  |  |  |  | **Male**  **n (%)** | **Female**  **n (%)** | **Male**  **n (%)** | **Female n (%)** |  |  |  |  |
| Golbranson et al. (35) | 1968 | USA | Prospective case series | 62 | NA | 17 (89.5) | 2 (10.5) | NA | NA | 10 (52.6%) | NA | 19 (100%) | NA |
| Condon et al. (33) | 1969 | USA | Prospective case series | 64, age range: 36-78 | NA | 29 (78.4) | 8 (21.6) | NA | NA | 21 (56.8%) | NA | 37 (100%) | NA |
| Mooney et al. (41) | 1976 | USA | Retrospective case series | Median age in diabetics: 60.5, median age in arteriosclerotic patients 63.0 | NA | 87 (45.8) | 103 (54.2) | NA | NA | 126 (66.3%) | NA | 64 (33.7%) | NA |
| Folsom et al. (42) | 1992 | USA | Retrospective cohort study | 64.6 | Not analysed | 56 (86.2) | 9 (13.8) | NA | NA | 39 (59%) | NA | 24 (35%) | NA |
| Schon et al. (37) | 2002 | USA | Prospective cohort study, with a retrospective control group | 53 years (range 23–79) | 54 years (range 33–90) | 18 (60.0) | 12 (40.0) | 16 (69.6) | 7 (30.4) | 17 (57%) | 12 (52%) | 11 (37%) | 5 (22%) |
| Woodburn et al. (40) | 2004 | UK | Randomised control trial | Not reported | Not reported | Both groups (RD + SD): 114 (74.0) | Both groups (RD + SD): 40 (26.0) | NA | NA | NA | NA | 56 (100%) | 56 (100%) |
| VanVelzen et al. (39) | 2005 | NL | Retrospective case control study | Immediate Fitting: 72.2 ± 11.3  Delayed Fitting: 75.5 ± 7.1 | 71.9 ± 10.2 | Immediate Fitting: 31 (54.4)  Delayed Fitting: 25 (62.5) | Immediate Fitting: 26 (45.6)  Delayed Fitting: 15 (37.5) | 32 (61.5) | 20 (38.5) | Immediate Fitting: 33 (57.9%)  Delayed Fitting: 22 (55%) | 40 (57.7%) | 97 (100%) | 52 (100%) |
| Ali et al. (34) | 2013 | USA | Retrospective cohort study | 61.5 | 69 | 31 (83.8) | 6 (16.2) | 24 (68.6) | 11 (31.4) | 33 (89.2%) | 26 (74.3%) | NA | NA |
| Sarkar et al. (36) | 2023 | USA | Retrospective cohort study | 57.4 | 63.0 | 44 (81.5) | 10 (18.5) | 25 (61.0) | 16 (39.0) | 36 (66.7%) | 25 (61.0%) | 34 (63.0%) | 32 (78.0%) |
| Bikk et al. (38) | 2024 | USA | Retrospective cohort study | 63.71 ± 11.16 | 66.9 ± 10.2 | 70 (100) | 0 (0) | 51 (98.1) | 1 (1.9) | 57 (49.13%) | 45 (38.8%) | 48 (68.6%) | 39 (75%) |

**Legend:** *Age reported as mean unless otherwise specified (e.g. median or range as reported in original studies). RD = rigid dressing; SD = standard dressing; DM: Diabetes mellitus; PVD = Peripheral vascular disease; NL = Netherlands.*

**Table 3:** *Clinical outcomes stratified by dressing type. Includes SSI rates, wound dehiscence, return to theatre, and mortality*

| **First Author** | **RD: Sample Size (n)** | **SD: Sample size (n)** | **RD: SSI rate (n (%))** | **SD: SSI rate (n (%))** | **RD: Wound Dehiscence Rate (n (%))** | **SD: Wound Dehiscence Rate (n (%))** | **RD: Return to Theatre (n (%))** | **SD: Return to Theatre (n (%))** | **RD: Mortality (n %))** | **SD: Mortality (n, (%))** |
| --- | --- | --- | --- | --- | --- | --- | --- | --- | --- | --- |
| Golbranson et al. (35) | 20 | 0 | 5 (25%) | NA | 2 (10%) | NA | 1 (5%) | NA | 7 (35%) | NA |
| Condon et al. (33) | 38 | 0 | 1 (2.6%) | NA | NA | NA | 7 (18.4%) | NA | 4 (10.5%) | NA |
| Mooney et al. (41) | 190 | 0 | 16 (8.4%) | NA | NA | NA | 8 (4.2%) | NA | 1 (0.5%) | NA |
| Folsom et al. (42) | 69 | 98 | 2 (2.9%) | NA | NA | NA | 1 (1.5%) | NA | 4 (5.8%) | NA |
| Schon et al. (37) | 30 | 23 | 0 | 4 (17.4%) | 0 | 3 (13%) | 0 | 10 (43.5%) | NA | NA |
| Woodburn et al. (40) | 56 | 56 | 12 (21.4%) | 10 (17.9%) | NA | NA | 2 (2.6%) | 3 (5.4%) | 14 (25.0%) | 10 (17.9%) |
| VanVelzen et al. (39) | 97 (IF: 57, DF: 40) | 52 | Superficial wound problems: 30 (30.9%), IF: 20 (35.1%), DF:10 (25%)  Deep wound problems: 24 (24.7%), IF: 10 (17.5%), DF: 14 (35%) | Superficial wound problems: 12 (23.1%)  Deep wound problems: 18 (34.6%) | NA | NA | Overall 13 (13%), IF: 3 (5.3%), DF: 10 (25.0%) | 9 (17.3%) | Overall: 29 (29.9%), IF: 16 (28.1%), DF: 13 (32.5%) | 12 (23.1%) |
| Ali et al. (34) | 37 | 35 | 7 (18.9%) | 9 (25.7%) | 29.70% | 25.00% | 5.4% | 27.6% | NA | 1 (2.9%) |
| Sarkar et al. (36) | 54 | 41 | 42 (77.8%) | 27 (65.9%) | NA | NA | 5 (9.3%) | 17 (41.5%) | NA | NA |
| Bikk et al. (38) | 70 | 52 | 7 (10%) | 7 (13.5%) | 5 (7.1%) | 12 (23%) | 5 (7.1%) | 5 (9.6%) | NA | NA |

**Legend:** *IF = immediate fitting; DF = delayed fitting; RD = rigid dressing; SD = standard dressing.*

**Table 4:** *Summary of findings: rigid versus soft dressings for major lower limb amputation*

| **Outcomes** | **No. of participants (studies)** | **Study design** | **Summary of findings** | **Quality of the evidence (GRADE)** | **Comments** |
| --- | --- | --- | --- | --- | --- |
| SSI rates | 710 (6 studies) | 1 RCT, 5 cohort studies, 1 case control study | We are uncertain whether rigid dressings reduce surgical site infection rates compared to soft dressings. | Very low^a,b,c,d^ | Substantial heterogeneity in SSI definitions, timing, and diagnostic criteria across studies. |
| Wound dehiscence | 247 (3 studies) | 3 cohort studies | We are uncertain whether rigid dressings reduce wound dehiscence compared to soft dressings. | Very low ^a,b,c,d^ | Small sample sizes and inconsistent definitions; baseline comorbidities may have influenced results. |
| Re-operation | 603 (6 studies) | 1 RCT, 4 cohort studies, 1 case control study | We are uncertain whether rigid dressings reduce the need for re-operation compared to soft dressings. | Very low ^a,b,c,d^ | Reported re-operation rates varied across studies; differences in study design and definitions of re-operation limit comparability and interpretation. |
| Mortality | 333 (3 studies) | 1 RCT, 1 cohort studies, 1 case control study | We are uncertain whether rigid dressings affect mortality compared to soft dressings. | Very low ^a,b,c,d^ | Inconsistent reporting and lack of standardised follow-up; no adjustment for baseline comorbidities. |

**Legend:** *RD = rigid dressing; SD = soft dressing.*

**Footnotes:**

1. Downgraded for risk of bias due to the predominance of observational studies and high or critical risk of bias in included studies.
2. Downgraded for inconsistency due to variability in effect estimates and heterogeneity in outcome reporting across studies.
3. Downgraded for indirectness due to differences in outcome definitions, measurement methods, and lack of standardisation across studies.
4. Downgraded for imprecision due to small sample sizes, low event numbers, and wide variation in reported outcomes.

**Figure 2:** *Risk of bias assessment of the included RCT risk using the RoB 2 tool*


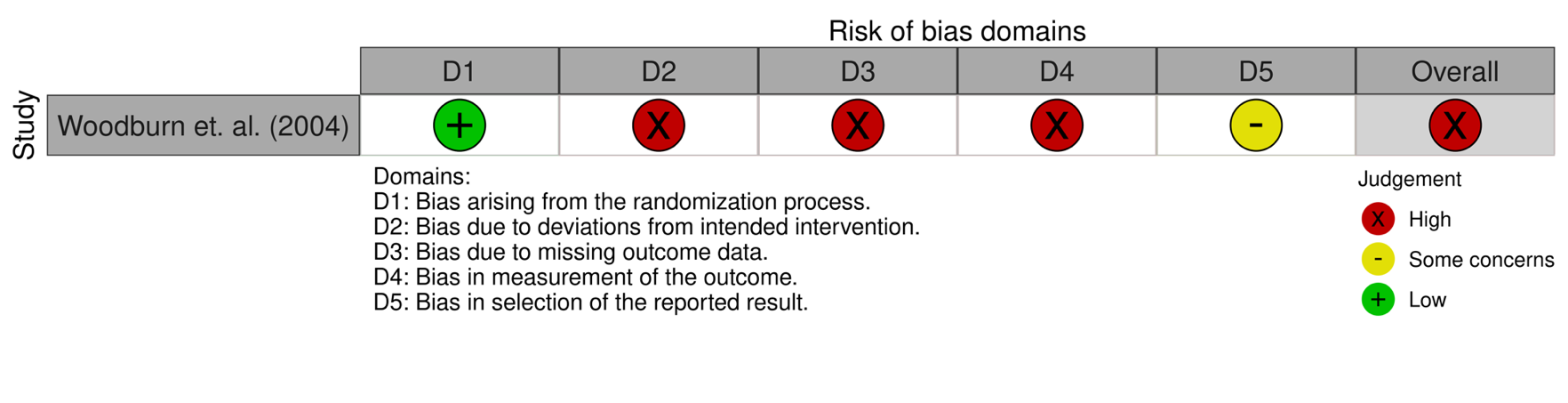


**Figure 3:** *Risk of bias assessment of included observational studies using ROBINS-I tool*


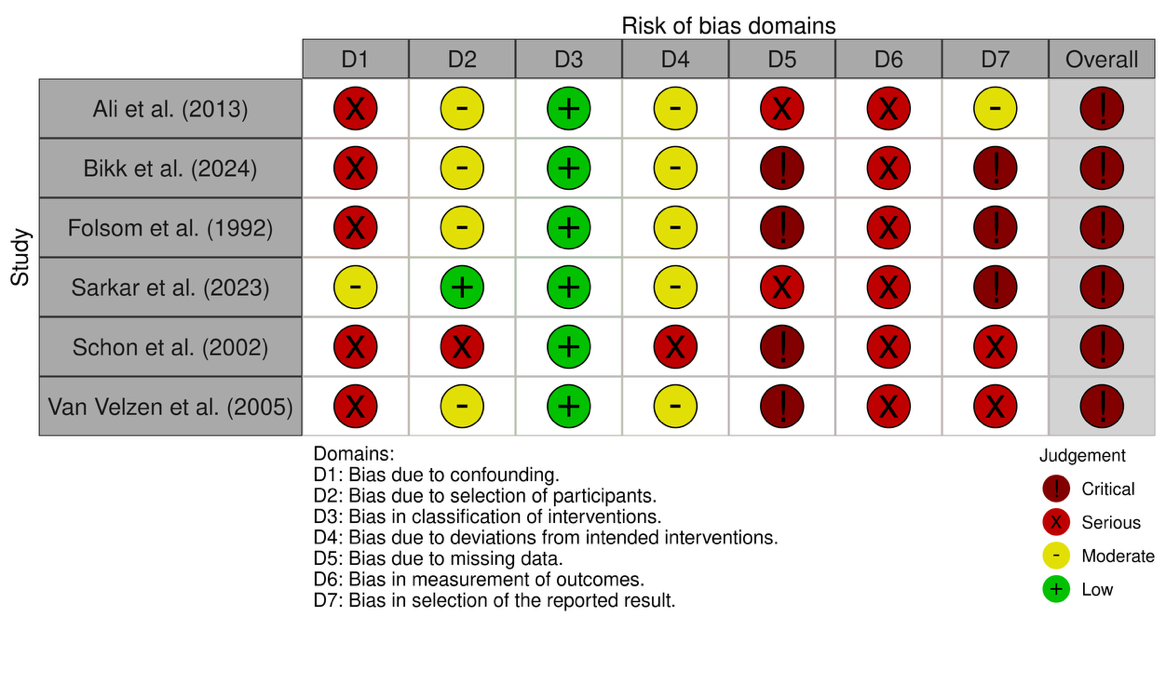


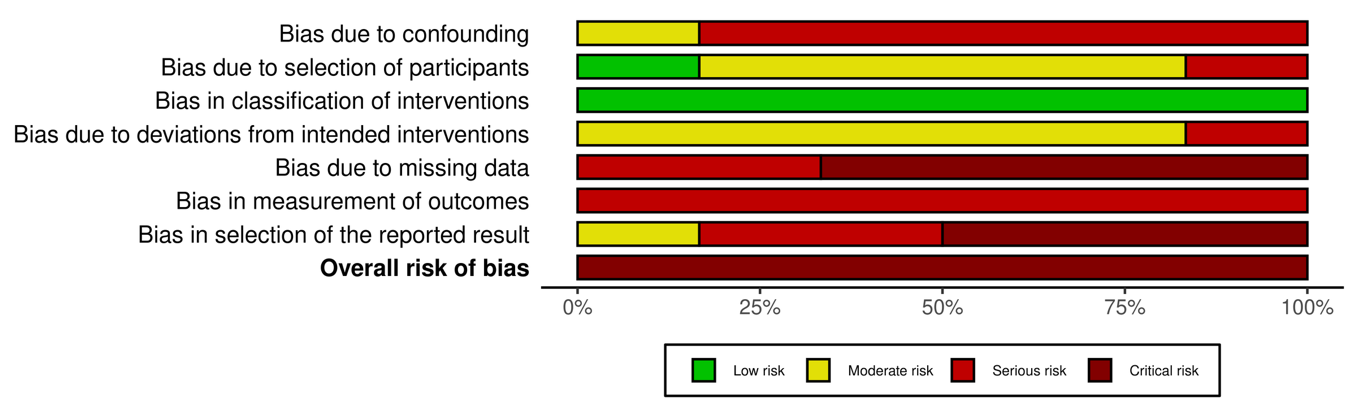


**Figure 4:** *Risk of bias assessment of included case series using a modification of the Joanna Briggs Institute critical appraisal tool*

| Study | Inclusion criteria | Condition measurement | Valid identification methods | Consecutive inclusion | Complete inclusion | Demographics reported | Clinical information reported | Outcomes reported | Site/clinic information | Appropriate statistical analysis | Overall risk of bias |
| --- | --- | --- | --- | --- | --- | --- | --- | --- | --- | --- | --- |
| Condon et. al. (1969) | Y | N | N | Y | Y | unclear | unclear | Y | N | Y | High |
| Mooney et. al. (1976) | unclear | N | N | Y | Y | unclear | unclear | Y | N | Y | High |
| Golbranson et. al. (1968) | N | unclear | unclear | unclear | unclear | unclear | unclear | N | N | Y | High |


**Legend:** *Y = yes; N = no; unclear = unclear; NA = not applicable. Column headings are abbreviated; full quality assessment questions are provided below.*

- **Inclusion criteria:** Were there clear criteria for inclusion in the case series? (Y/N/unclear/NA)
- **Condition measurement:** Was the condition measured in a standard, reliable way for all participants included in the case series? (Y/N/unclear/NA)
- **Valid identification methods:** Were valid methods used for identification of the condition for all participants included in the case series? (Y/N/unclear/NA)
- **Consecutive inclusion:** Did the case series have consecutive inclusion of participants? (Y/N/unclear/NA)
- **Complete inclusion:** Did the case series have complete inclusion of participants? (Y/N/unclear/NA)
- **Demographics reported:** Was there clear reporting of the demographics of the participants in the study? (Y/N/unclear/NA)
- **Clinical information reported:** Was there clear reporting of clinical information of the participants? (Y/N/unclear/NA)
- **Outcomes reported:** Were the outcomes or follow-up results of cases clearly reported? (Y/N/unclear/NA)
- **Site/clinic information:** Was there clear reporting of the presenting site(s)/clinic(s) demographic information? (Y/N/unclear/NA)
- **Statistical analysis:** Was statistical analysis appropriate? (Y/N/unclear/NA)
